# Dynamics of peripheral immune cells and their HLA‐G and receptor expressions in a patient suffering from critical COVID‐19 pneumonia to convalescence

**DOI:** 10.1002/cti2.1128

**Published:** 2020-05-10

**Authors:** Sheng Zhang, Jun Gan, Bao‐Guo Chen, Dan Zheng, Jian‐Gang Zhang, Rong‐Hai Lin, Yi‐Ping Zhou, Wei‐Ying Yang, Aifen Lin, Wei‐Hua Yan

**Affiliations:** ^1^ Department of Intensive Care Unit TaiZhou Hospital of Zhejiang Province Wenzhou Medical University LinHai Zhejiang China; ^2^ Medical Research Center TaiZhou Hospital of Zhejiang Province Wenzhou Medical University LinHai Zhejiang China; ^3^ Biological Resource Center TaiZhou Hospital of Zhejiang Province Wenzhou Medical University LinHai Zhejiang China

**Keywords:** COVID‐19, HLA‐G, peripheral immune cells, SARS‐CoV‐2

## Abstract

**Objectives:**

Host immune responses are indispensable to combat the disease. We report the dynamics of peripheral immune cells, cytokines, and human leucocyte antigen‐G (HLA‐G) and its receptor expressions in a patient suffering from critical COVID‐19 pneumonia to convalescence.

**Methods:**

Clinical data of the patient were collected from medical records. The expressions of HLA‐G and receptors ILT2, ILT4 and KIR2DL4 in peripheral immune cells were measured with flow cytometry.

**Results:**

From critical COVID‐19 to the convalescent stage, early lymphopenia was improved (median: 0.6 × 10^9^ L^−1^ vs. 0.9 × 10^9^ L^−1^, *P* = 0.009), and an obvious fluctuation in WBC and neutrophil counts was observed. Initially, low levels of CD4^+^ T cells (from 120 to 528 μL^−1^) and CD8^+^ T cells (from 68 to 362 μL^−1^) gradually increased to normal levels. Meanwhile, high IL‐6 (from 251.8 to 6.32 pg mL^−1^), IL‐10 (from 39.53 to 5.21 pg mL^−1^) and IFN‐γ (from 13.55 to 3.16 pg mL^−1^) levels decreased, and IL‐4 (from 2.36 to 3.19 pg mL^−1^) and TNF‐α (from 2.27 to 20.2 pg mL^−1^) levels increased quickly when the viral RNA returned negative. Moreover, the percentage of HLA‐G^+^ T cells, B cells and monocytes follows high–low–high pattern, while the percentage of receptors ILT2‐, ILT4‐ and KIR2DL4‐expressing cells remained relatively stable.

**Conclusion:**

Our findings provide valuable information on the dynamics of early peripheral immunological responses in SARS‐CoV‐2 infection. CD4^+^ and CD8^+^ T cells, cytokines and HLA‐G^+^ immune cells are associated with the natural history of the critical COVID‐19 patient; however, future studies are necessary.

## Graphical abstract

Pneumonia COVID‐19 is threatening public health. Host immune responses are indispensable to combat the disease. In this paper, we report on the dynamics of early CD4^+^ and CD8^+^ T cells, cytokines and HLA‐G^+^ immune cells that are associated with the natural history of a critical COVID‐19 patient.

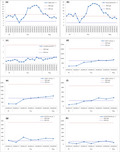

## Introduction

An ongoing outbreak of pneumonia COVID‐19 caused by the RNA virus SARS‐CoV‐2 (initially 2019‐nCoV) is threatening public health.^1^ Since the first reported case on 31 December 2019 to 27 February 2020, more than 2700 cases have resulted in death, and an increasing number of patients with COVID‐19 have been consecutively reported in more than twenty countries including Japan, Singapore, Thailand, Korea and other nations.^2^ In addition to the outbreak of severe acute respiratory syndrome‐related coronavirus (SARS‐CoV) in 2002 and the Middle East respiratory syndrome‐related coronavirus (MERS‐CoV) in 2012, SARS‐CoV‐2 has become the third coronavirus that seriously threatens public health in the past two decades. The World Health Organization (WHO) has announced the outbreak of SARS‐CoV‐2‐ caused COVID‐19 as a Public Health Emergency of International Concern (PHEIC).^3^, ^4^

Currently, no effective therapeutic agents are available for SARS‐CoV‐2, although many pioneer clinical trials are underway. It has been found that host humoral and cellular antiviral immune responses are indispensable to fight back and control infectious diseases.^5^ Immune functional modulators and effectors such as cytokines and chemokines, CD4^+^ and CD8^+^ T cells, and human leucocyte antigen (HLA) expression are interfered with via viral infection and can play crucial roles in the control of virus replication and the outcome of patients.^6^ In this scenario, human leucocyte antigen‐G (HLA‐G) and its immune cell surface‐expressed receptor signalling pathway has been well known to modulate the functions of T cells, B cells and NK cells, and is involved in viral infection.^7^, ^8^

In this study, we recorded and analysed the dynamics of peripheral immune cells, the expression of HLA‐G and its receptors ILT2, ILT4 and KIR2DL4 in peripheral immune cells, and the outcomes of a patient infected with SARS‐CoV‐2 (critical COVID‐19) during the 23‐day hospitalisation. These findings were compared between the day when SARS‐CoV‐2 RNA confirmed positive and the day when the result returned to negative. Our preliminary data may help future studies on SARS‐CoV‐2 infection.

## Results

### Laboratory data and cytokine profiles

The record of the complete blood counts and serum/plasma chemical laboratory tests were available from the first day of hospitalisation on 19 January 2020 to the day the patient was discharged from ICU on 12 February 2020 (a period of 23 days) when the disease was improved to convalescence. Baseline characteristics and the medical history of the patient are detailed in Table 1.

**Table 1:**
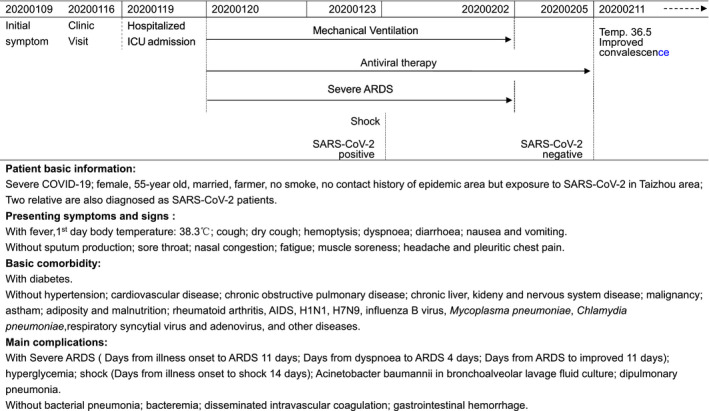
Baseline characteristics of the patient infected with SARS‐CoV‐2

**Figure 1:**
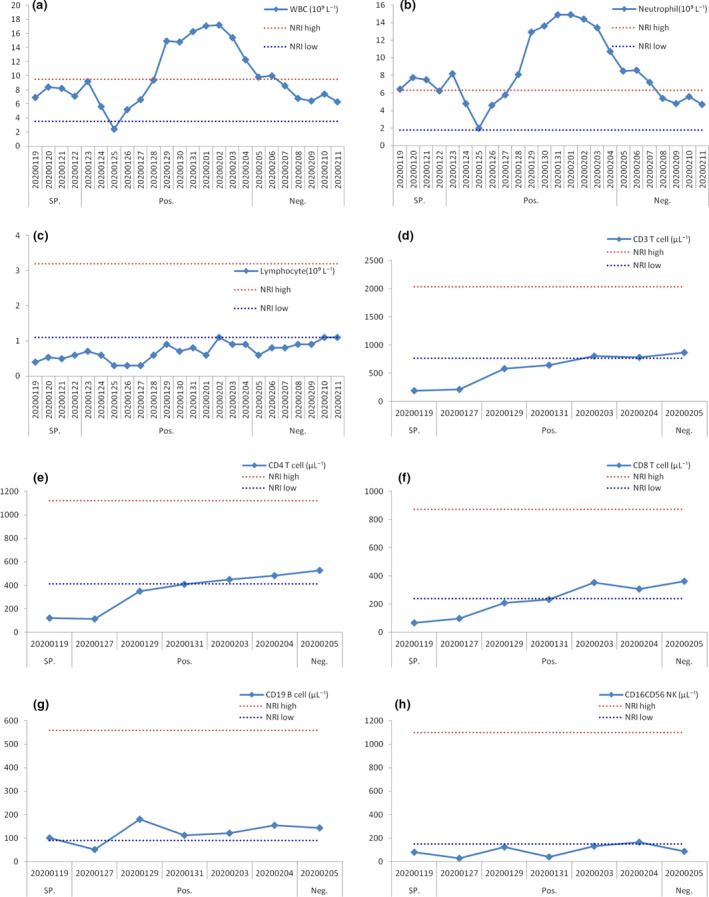
Dynamics of the peripheral immune cell counts in the critical COVID‐19 patient during hospitalisation. WBC counts **(a)**, neutrophil **(b)**, lymphocyte **(c)**, CD3^+^ T lymphocytes **(d)**, CD4^+^ T lymphocytes **(e)**, CD8^+^ T lymphocytes **(f)**, B lymphocytes **(g)** and NK cells **(h)**. WBC, white blood cells; NRI, normal reference interval; SP., suspected SARS‐CoV‐2 infection; Pos., SARS‐CoV‐2 RNA positive; Neg., SARS‐CoV‐2 RNA returned to negative. Blue and red dashes indicate the lower and upper values of the normal reference interval, respectively.

Laboratory results showed the WBC count (median: 8.5 × 10^9^ L^−1^; range: 2.4 × 10^9^ to 17.2 × 10^9^ L^−1^) and neutrophil count (median: 7.64 × 10^9^ L^−1^; range: 2.0 × 10^9^ to 14.9 × 10^9^ L^−1^) fluctuated in three phases, from normal to higher, and returned to normal levels. The initial lymphopenia gradually improved and increased to normal but was at low level during the period (median: 0.6 × 10^9^ L^−1^ vs. 0.9 × 10^9^ L^−1^, *P* = 0.009). Among the subpopulations of lymphocytes, the initial low levels of both CD4^+^ T cells (from 120 to 528 μL^−1^) and CD8^+^ T cells (from 68 to 362 μL^−1^) slowly increased to normal range, which might contribute to the normalisation of the total CD3^+^ T cells (from 192 to 869 μL^−1^). However, B cells (from 100 to 143 μL^−1^) and NK cells (from 80 to 88 μL^−1^) were observed to be more stable and remained at a lower level. Similar results were observed when these data were compared between the SARS‐CoV‐2‐positive and SARS‐CoV‐2‐negative status in which the lymphocyte and monocyte counts increased, while the WBC count (median: 9.2 × 10^9^ L^−1^ vs. 7.1 × 10^9^ L^−1^) and neutrophil count (median: 8.1 × 10^9^ L^−1^ vs. 5.5 × 10^9^ L^−1^) decreased to normal levels (Figure 1). Fluctuations in hepatic and renal function biomarkers were observed during hospitalisation and decreased when the virus returned to negative (Table 2).

**Table 2:** Comparison of laboratory data between SARS‐CoV‐2‐positive and SARS‐CoV‐2 returned to negative status

Laboratory test	Reference range	SARS‐CoV‐2 Pos. Median (range)	SARS‐CoV‐2 Neg. Median (range)	*P‐*value
White blood cell count (10^9^ L^−1^)	3.5–9.5	9.2 (2.4–17.2)	7.1 (6.2–10.0)	0.238
Neutrophil count (10^9^ L^−1^)	1.8–6.3	8.1 (2.0–14.9)	5.5 (4.3–8.6)	0.075
Lymphocyte count (10^9^ L^−1^)	1.1–3.2	0.6 (0.3–1.1)	0.9 (0.6–1.3)	0.009
Mononuclear count (10^9^ L^−1^)	0.1–0.6	0.2 (0.0–1.1)	0.35 (0.2–0.5)	0.374
Red blood cell count (10^12^ L^−1^)	4.3–5.8	3.61 (3.23–4.3)	3.05 (2.75–3.16)	<0.001
Platelet count (10^9^ L^−1^)	125–350	239 (125–363)	186 (110–209)	0.262
Neutrophil (%)	40–75	87.2 (82.3–92.5)	77.4 (69.7–86.6)	<0.001
Lymphocyte (%)	20–50	6.1 (3.6–12.6)	13.45 (6.6–20.9)	<0.001
Monocyte (%)	3–10	4.2 (0.0–7.8)	4.7 (3.8–5.8)	0.406
C‐reactive protein (mg L^−1^)	<8.0	32.8 (6.7–208.4)	53.4 (4.3–79.2)	0.711
K^+^ (mmol L^−1^)	3.4–4.5	4.1 (3.5–4.72)	3.425 (3.0–4.79)	0.023
Na^+^ (mmol L^−1^)	136–146	137.5 (133–143)	137.95 (134–141.3)	0.842
Cl^−^ (mmol L^−1^)	98–106	97 (91.3–116)	98.15 (94.6–103)	0.628
Ca^+^ (mmol L^−1^)	1.15–1.29	2.02 (1.04–2.39)	2.25 (1.09–2.29)	0.977
Creatinine (μmol L^−1^)	59–104	50 (42–62)	46.5 (43–53)	0.494
Urea (mmol L^−1^)	3.1–8.0	10.1 (3.46–12.12)	7.69 (6.01–9.78)	0.023
Glucose (mmol L^−1^)	3.9–6.1	9.06 (7.05–15.4)	9.78 (7–13)	0.628
Prothrombin time (s)	11–14.5	13.6 (13–14)	13.45 (13.1–14)	0.785
Thrombin time (s)	14–21	16.2 (13.7–19.2)	16.15 (13.4–16.7)	0.703
Activated partial thromboplastin time (s)	28–42	31.2 (25.6–45.3)	34.65 (34–36.9)	0.412
Fibrinogen (g L^−1^)	2–4	3.36 (1.88–6.99)	5.56 (4.75–6.52)	0.163
D‐dimer (mg L^−1^)	0–0.05	2.29 (1.82–20)	1.08 (1.05–1.83)	0.011
Procalcitonin (ng mL^−1^)	<0.05	0.09 (0.05–0.39)	0.11 (0.08–0.14)	0.780
Aspartate aminotransferase (U L^−1^)	15–40	31(15–71)	19 (15–21)	0.033
Alanine aminotransferase (U L^−1^)	9–50	33 (17–49)	17.5 (14–25)	0.005
Alkaline phosphatase (U L^−1^)	45–125	54 (40–71)	70 (59–74)	0.002
γ‐Glutamyl transpeptidase (U L^−1^)	10–60	68 (28–80)	45 (40–55)	0.041
Total bilirubin (mmol L^−1^)	3.4–20.5	11.4 (6.1–17.9)	6.2 (5.3–11.2)	0.006
Direct bilirubin (mmol L^−1^)	0–6.8	4.15 (2.4–5.4)	1.95 (1.3–3.2)	<0.001
Indirect bilirubin (mmol L^−1^)	2.0–16.5	7.85 (3.4–13)	4.2 (3.4–8)	0.033
Total protein (g L^−1^)	65–85	71.8 (63–74.2)	67.55 (64.5–69.6)	0.179
Albumin (g L^−1^)	40–55	41.45 (23–47.2)	38.6 (36.7–43.3)	0.547
Globulin (g L^−1^)	20–40	28.85 (26.2–41.7)	28.05 (26.3–30.7)	0.659
α–L‐fucosidase (U L^−1^)	0–40	37 (30–44)	31 (28–35)	0.045
Triglyceride (mmol L^−1^)	0.56–1.70	3.39 (1.35–5.1)	2.72 (1.59–3.94)	0.180
Total cholesterol (mmol L^−1^)	2.84–5.69	6.25 (3.17–7.51)	5.32 (4.95–5.73)	0.377
High‐density lipoprotein (mmol L^−1^)	0.9–2.19	0.83 (0.37–1)	0.93 (0.88–0.96)	0.019
Low‐density lipoprotein (mmol L^−1^)	<3.36	3.99 (1.92–4.51)	3.27 (3.07–3.87)	0.510
Apolipoprotein A I (g L^−1^)	1.2–1.8	0.79 (0.66–1.18)	0.99 (0.97–1.18)	0.164
Apolipoprotein B (g L^−1^)	0.6–1.14	1.5 (0.88–1.83)	1.29 (1.26–1.33)	0.730
Lipoprotein A (mg L^−1^)	<300	134 (80–331)	338.5 (251–366)	0.024

**Figure 2:**
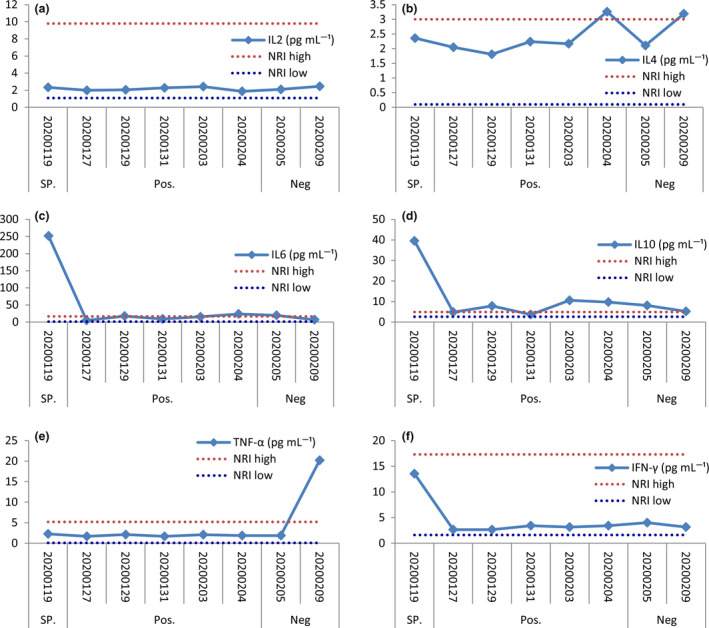
Dynamics of cytokine levels in the critical COVID‐19 patient during hospitalisation. Levels of IL‐2 **(a)**, IL‐4 **(b)**, IL‐6 **(c)**, IL‐10 **(d)**, TNF‐α **(e)** and IFN‐γ **(f)**. IL, interleukin; TNF, tumor necrosis factor; IFN, interferon; NRI, normal reference interval. SP., suspected SARS‐CoV‐2 infection; Pos., SARS‐CoV‐2 RNA positive; Neg., SARS‐CoV‐2 RNA returned to negative. Blue and red dashes indicate the lower and upper values of the normal reference interval, respectively.

Plasma cytokine analysis showed that the patient’s initial higher levels of IL‐6 (from 251.8 to 6.32 pg mL^−1^), IL‐10 (from 39.53 to 5.21 pg mL^−1^) and IFN‐γ (from 13.55 to 3.16 pg mL^−1^) decreased rapidly, and IL‐4 (from 2.36 to 3.19 pg mL^−1^) and TNF‐α (from 2.27 to 20.2 pg mL^−1^) increased quickly when the viral RNA was returned as negative; however, the levels of IL‐2 (from 2.34 to 2.36 pg mL^−1^) were more stable (Figure 2).

### HLA‐G and receptor expressions in peripheral immune cells

**Figure 3:**
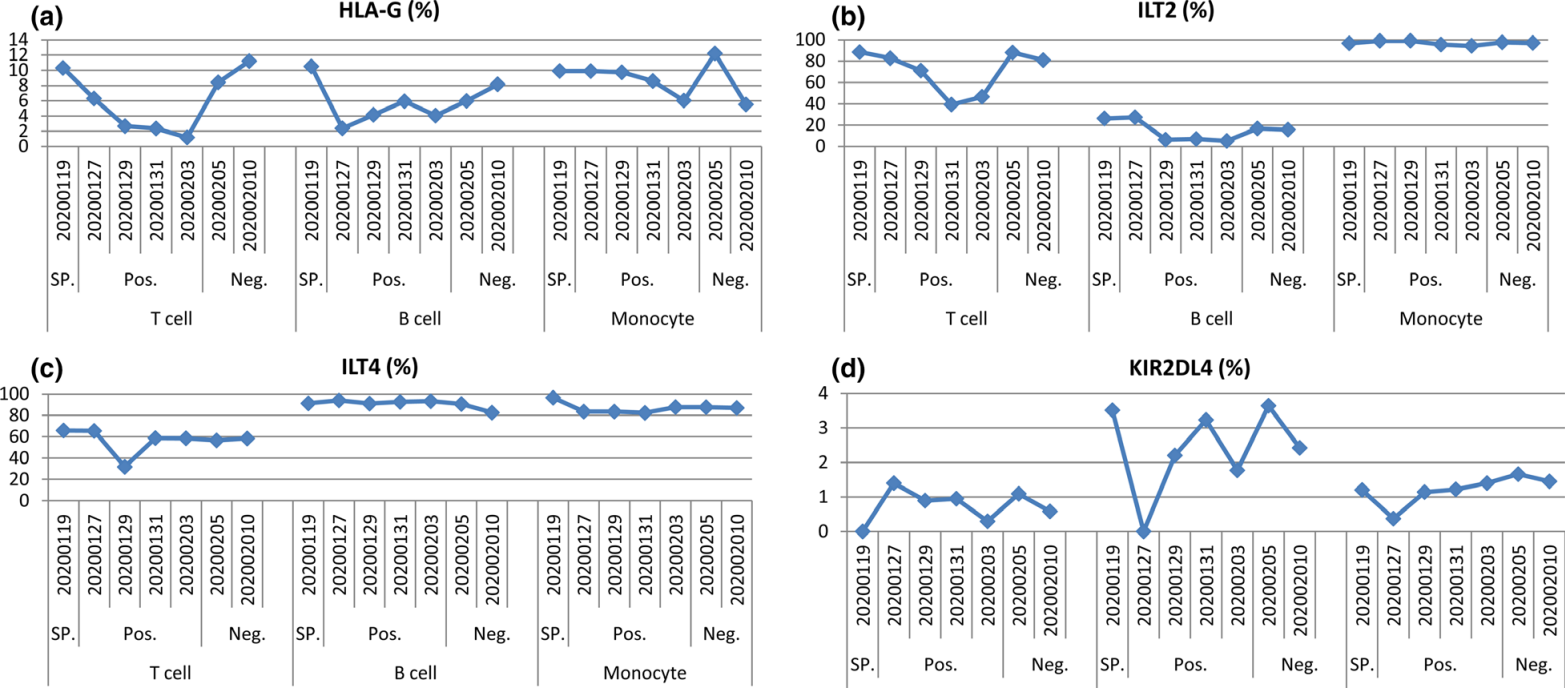
Dynamics of the percentage of HLA‐G **(a)**, and receptors ILT2 **(b)**, ILT4 **(c)**, and KIR2DL4 **(d)** in peripheral T cells, B cells and monocytes of the critical COVID‐19 patient during hospitalisation. SP., suspected SARS‐CoV‐2 infection; Pos., SARS‐CoV‐2 RNA positive; Neg., SARS‐CoV‐2 RNA returned to negative.

The percentage of HLA‐G^+^ T cells (median: 6.29%; range: 1.18–11.2%), B cells (median: 5.93%; range: 2.38–10.50%) and monocytes (median: 9.73%; range: 5.51–12.20%) is of a high–low–high pattern, while the percentage of receptors ILT2‐, ILT4‐ and KIR2DL4‐expressing cells remained more stable. The percentage of the ILT2^+^ T cells, B cells and monocytes was with the median of 80.90% (range: 39.3–88.4%), 15.80% (range: 5.18–27.40) and 97.0% (range: 94.30–99.10%), respectively; that of the ILT4^+^ T cells, B cells and monocytes was with the median of 58.5% (range: 37.1–65.8%), 91.2% (range: 82.5–94.0%) and 86.9% (range: 82.3–96.5%), respectively; and that of the KIR2DL4^+^ T cells, B cells and monocytes was with the median of 0.89% (range: 0.0–1.40), 2.42% (range: 0.0–3.64%) and 1.22% (range: 0.37–1.66%), respectively (Figure 3).

**Figure 4:**
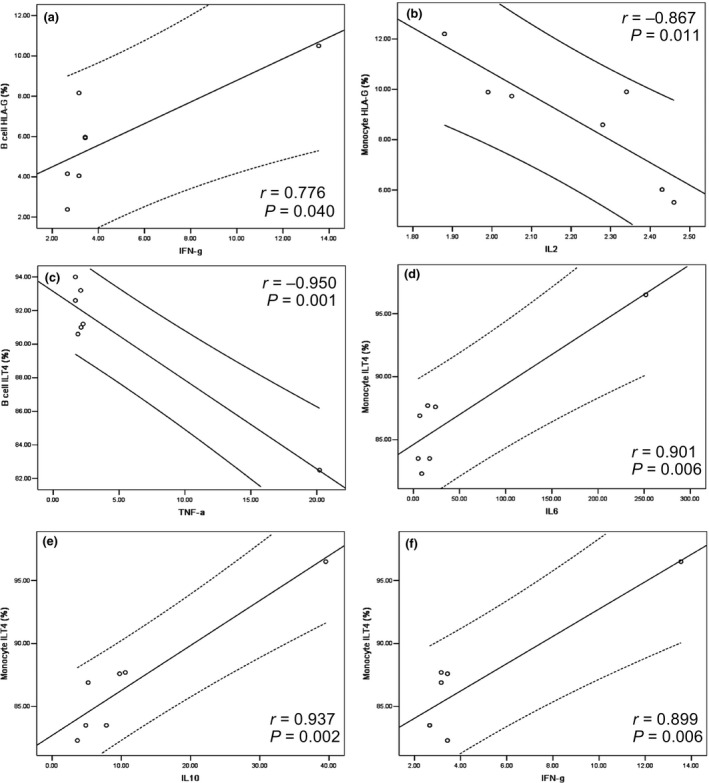
Correlation between the percentage of HLA‐G and the receptor expression of immune cells and cytokine levels. Correlation between HLA‐G^+^ B cells and IFN‐γ **(a)**; HLA‐G^+^ monocytes and IL‐2 **(b)**; ILT4^+^ B cells and TNF‐α **(c)**; and ILT4^+^ monocytes and IL‐6 **(d)**, IL‐10 **(e)** and IFN‐γ **(f)**.

Previous studies have revealed that cytokines can modulate HLA‐G and its receptor expressions. In this preliminary study, we analysed the relationship between the levels of cytokines and HLA‐G and its receptor expressions in peripheral immune cell subsets. HLA‐G expression in B cells was positively related to IFN‐γ levels (*r* = 0.776, *P* = 0.040) while HLA‐G expression in monocytes was negatively related to IL‐2 levels (*r* = −0.867, *P* = 0.011). For receptor expression, we found that only ILT4 expression was strongly related to the cytokines. ILT4 expression in B cells was negatively correlated with TNF‐α levels (*r* = 0.950, *P* = 0.001), while ILT4 expression in monocytes was positively correlated with the cytokines including IL‐6 (*r* = 0.901, *P* = 0.006), IL‐10 (*r* = 0.937, *P*= 0.002) and IFN‐γ (*r* = 0.899, *P* = 0.006) (Figure 4).

## Discussion

The novel coronavirus SARS‐CoV‐2 identified in late December 2019 is currently threatening public health and has become a major concern worldwide. The accumulation of confirmed patients is still on the rise, though the daily increase in cases is declining.^2^ Although the epidemiological features such as the transmission, spreading models and clinical spectrum have become clearer, our understanding of the SARS‐CoV‐2 infection is limited.^9^, ^10^ Unfortunately, no effective and powerful therapeutic agents are available to combat the novel SARS‐CoV‐2, and supportive treatment is currently commonly employed. In this scenario, host antiviral immune status, including innate and adaptive immune responses, has been demonstrated to play critical roles in virus clearance and the outcome of infectious diseases.^11^

In this study, we report a full spectrum, mainly focused on the dynamics of peripheral immune cells, the expressions of HLA‐G and its receptors (ILT2, ILT4 and KIR2DL4) in peripheral immune cells, and the outcomes of a patient infected with SARS‐CoV‐2 (critical COVID‐19) during the 23‐day hospitalisation. Lymphopenia is commonly observed in patients with COVID‐19. In a cohort of 41 patients, Huang *et al*.^12^ reported that 63% (26/42) of COVID‐19 patients had lymphopenia, and the rate was markedly higher in patients admitted to the ICU than in non‐ICU patients (85% vs. 54%). In the first US‐confirmed cases of SARS‐CoV‐2‐infected patients, the laboratory data by Holshue *et al*.^13^ showed that from the third to tenth hospital day, the lymphocyte count was at a low level of 1070/μL and had gradually increased to 2100/μL along with the improved condition of the disease. In line with these reports, our data showed that the lymphocyte count was also observed to have increased near to the normal level at the moment of disease improvement. Notably, our data also showed that the increased lymphocyte count might be contributed by the increase in both CD4^+^ and CD8^+^ T cells. However, B cells and NK cells remain more stable at rather low levels. T cells are critical for the host’s cellular immune response against viral infection. The subpopulation of CD8^+^ cytotoxic T lymphocytes (CTLs) can kill virus‐infected cells directly, and CD4^+^ helper T lymphocytes are essential for B lymphocytes to produce virus‐specific antibodies.^14^ Recently, a study by Xu *et al*.^15^ revealed that significantly decreased but hyperactivated peripheral immune cells such as CD4^+^ and CD8^+^ T cells were observed in a COVID‐19 patient, and unfortunately, the patient died. The authors speculated that the hyperactivated immune cells might be involved in immune injury for the disease. However, the sampling day during the disease course for flow cytometry analysis was unclear, which is of importance for the peripheral immune cell response that could be associated with the disease status, as our present findings indicated. Consequently, the increase in both CD4^+^ and CD8^+^ T cells in this case, as previously reported in other infectious diseases, may play a similar antiviral role with respect to the improvement in the disease.^16^

Moreover, we found that the high levels of cytokines IL‐6, IL‐10 and IFN‐γ decreased rapidly, and the levels of IL‐4 and TNF‐α increase quickly when the viral RNA returned negative. In line with our observations, a previous study revealed that IL‐6 and IFN‐γ levels were dramatically elevated during the acute phase of MERS‐CoV infection, whereas the IL‐6 levels were observed to be related to disease severity.^17^ However, in contrast to our study, TNF‐α and IL‐10 were not detected in patients with MERS‐CoV infection, indicating that different features inducing cytokine production might exist between the SARS‐CoV‐2 and MERS‐CoV infections.

The pathogenesis mechanisms of SARS‐CoV‐2 infection are yet unknown; however, they are thought to be similar to other viruses that have evolved various strategies, such as disturbing HLA expression and viral antigen presentation, which is commonly applied by viruses to escape the host immune system recognition and destruction.^18^ HLA‐G has been intensively investigated in viral infectious diseases.^19^ Most previous studies have demonstrated that HLA‐G expression could be upregulated in virus‐infected cells such as HIV, HCMV and HCV, and have speculated that the upregulation of HLA‐G expression is a potential strategy for virus immune evasion.^20^, ^21^, ^22^ The underlying mechanism is that HLA‐G can bind immune inhibitory receptors such as ILT2 and ILT4, and consequently inhibit the functions of immune cells and favor virus immune escape.^23^

Using the human lung epithelial cell line Calu‐3 and global transcriptomic profiling, Josset *et al*.^24^ demonstrated that two lethal coronaviruses MERS‐CoV and SARS‐CoV could induce predominant differences in host gene expression responses. Data showed that HLA genes, including HLA‐G, were observed to be specifically downregulated after MERS‐CoV infection, while these genes were upregulated after SARS‐CoV infection. In this study, our data showed that HLA‐G expression in peripheral immune cells such as T cells, B cells and monocytes follows a high–low–high pattern, which may reflect the three stages of infection, replication and clearance of SARS‐CoV‐2; however, receptors ILT2, ILT4 and KIR2DL4 remained relatively stable. Moreover, correlations between HLA‐G and its receptor expressions and cytokine production were observed in our study, such as different correlations between IFN‐γ and IL‐2 and HLA‐G expression in B cells and monocytes. The dynamics of HLA‐G expression in peripheral immune subsets from SARS‐CoV‐2‐positive to SARS‐CoV‐2‐negative indicated that the status of SARS‐CoV‐2 infection might be involved in the regulation of HLA‐G expression. Given that HLA‐G is an antigen presenter similar to other HLA I antigens, the downregulated HLA‐G expression by SARS‐CoV‐2 can impair the virus CD8^+^ CTL‐mediated recognition and support immune evasion.

However, our preliminary study has notable limitations. First, this study is based on only one critical COVID‐19 case, and real world of the HLA‐G and its receptor expressions in peripheral immune subsets in the COVID‐19 case is still unknown. Second, the outcomes only on several time points during the 23‐day hospitalisation were assayed; the variation of the expression of HLA‐G and its receptors, and the levels of cytokines may be underestimated. Third, the underlying mechanisms involved in dynamics of HLA‐G and its receptors, and the levels of cytokines are yet to be explored. Therefore, more detailed and larger cohorts of the disease need to be investigated.

In summary, the case we have studied shows the dynamics of the peripheral immunological responses during SARS‐CoV‐2 infection. However, future studies on the clinical significance of our findings are necessary.

## Methods

### Patient

A 55‐year‐old female patient visited Wenzhou Medical University Affiliated Taizhou Hospital of Zhejiang Province, China, on 16 January 2020, seven days after the onset of symptoms. The patient was hospitalised and admitted to the ICU on 19 January 2020, and the SARS‐CoV‐2 infection was confirmed on 23 January 2020 with the virus RNA RT‐PCR result (specimen from throat swab) when the RT‐PCR reagents (Shanghai BioGerm Medical Biotechnology Co., Ltd) for this virus were available. Before the SARS‐CoV‐2 infection confirmation, a panel of suspected viruses that could cause pneumonia were excluded, such as H1N1, H7N9, influenza B virus, *Mycoplasma pneumoniae*, *Chlamydia pneumoniae*, respiratory syncytial virus and adenovirus.

This patient is the first to be infected with SARS‐CoV‐2 in our hospital and diagnosed as the first critical COVID‐19 case in Zhejiang Province, who had been admitted to the ICU for 23 days (from 19 January 2020 to 11 February 2020). Fortunately, the disease improved to a convalescent stage.

This study was approved by the institutional ethics committee (#K20200111), and written consent was obtained from the patient.

### Data collection

Clinical charts, nursing records and laboratory findings were reviewed and recorded for this laboratory‐confirmed SARS‐CoV‐2‐infected patient from 19 January 2020 to 11 February 2020. By then, the condition of this critical COVID‐19 case was improved and moved to the general ward in the Public Health Center of Taizhou City. The epidemiological, clinical, laboratory, treatment and outcome data were reviewed and collected from electronic medical records.

### Cell surface markers and cytokine analysis by flow cytometry

EDTA anticoagulation peripheral blood was drawn from the patient for routine clinical laboratory tests. One hundred microlitres of whole blood and red cells was lysed by lysis buffer (BD FACS Lysing Solution; BD Biosciences, San Jose, CA, USA) and was used for flow cytometry analysis. HLA‐G, ILT2, ILT4 and KIR2DL4 levels on peripheral T cells, B cells and monocytes were determined. A representative flow cytometry analysis is shown in Supplementary figure 1. For T cells, two settings of antibody combinations were performed: anti‐CD3‐APC (BD Biosciences, San Diego, CA, USA) cells were probed with anti‐HLA‐G‐FITC (Exbio, Prague, Czech Republic) and anti‐ILT2‐PE (Biolegend, San Diego, CA, USA); and anti‐CD3‐APC (BD Biosciences, San Diego, CA, USA) cells were probed with anti‐KIR2DL4‐PE (Exbio) and anti‐ILT4‐PE‐CY7 (Biolegend). For B cells, anti‐CD19‐Per‐CP (BD, USA) cells were probed with anti‐HLA‐G‐FITC (Exbio), anti‐ILT2‐APC (Biolegend), anti‐ILT4‐PE‐CY7 (Biolegend) and anti‐KIR2DL4‐PE (Exbio). For monocytes, anti‐CD14‐APC‐CY7 (BD Biosciences, San Diego, CA, USA) cells were probed with anti‐HLA‐G‐FITC (Exbio), anti‐ILT2‐APC (Biolegend), anti‐ILT4‐PE‐CY7 (Biolegend) and anti‐KIR2DL4‐PE (Exbio). Lymphocyte subsets and cytokines IL‐2, IL‐4, IL‐6, IL‐10, TNF‐α and IFN‐γ were measured with a Cytometric Bead Array Th1/Th2 Subset Kit (Hangzhou Cell Gene Biotech Co., LTD, Hangzhou, China) according to the manufacturer’s instructions. Flow cytometry analysis was performed on a BD Canto II flow cytometer, and data were analysed using the BD FACSuite software (BD Biosciences, San Jose, CA, USA).

### Statistical analysis

Statistical analysis was performed using SPSS 13.0 software (SPSS, Inc., Chicago, IL, USA). Continuous numeric data were analysed using the Mann–Whitney *U*‐test. The correlations between variables were analysed using the Spearman test. A two‐sided *P*‐value < 0.05 was considered statistically significant.

## Conflict of interest

The authors declare no conflict of interest.

## Author contributions

Sheng Zhang: Data curation, Investigation, Resources, Validation. Jun Gan: Data curation, Funding acquisition, Investigation, Methodology. Bao‐Guo Chen: Data curation, Investigation, Methodology, Software. Dan Zheng: Data curation, Investigation, Resources. Jian‐Gang Zhang: Data curation, Resources. Rong‐Hai Lin: Data curation, Investigation, Resources. Yi‐Ping Zhou: Data curation, Investigation, Resources. Wei‐Ying Yang: Data curation, Investigation. Aifen Lin: Conceptualization, Data curation, Formal analysis, Funding acquisition, Validation, Writing‐original draft, Writing‐review & editing. Wei‐Hua Yan: Conceptualization, Data curation, Formal analysis, Funding acquisition, Software, Validation, Writing‐original draft, Writing‐review & editing.

## Supporting information

Supplementary figure 1
