# Supplementary figures and images for: Dynamics of peripheral immune cells and their HLA‐G and receptor expressions in a patient suffering from critical COVID‐19 pneumonia to convalescence
Source: Clin Transl Immunology. 2020 May 10;9(5):e1128. doi: 10.1002/cti2.1128 (PMC7211507; doi:10.1002/cti2.1128)

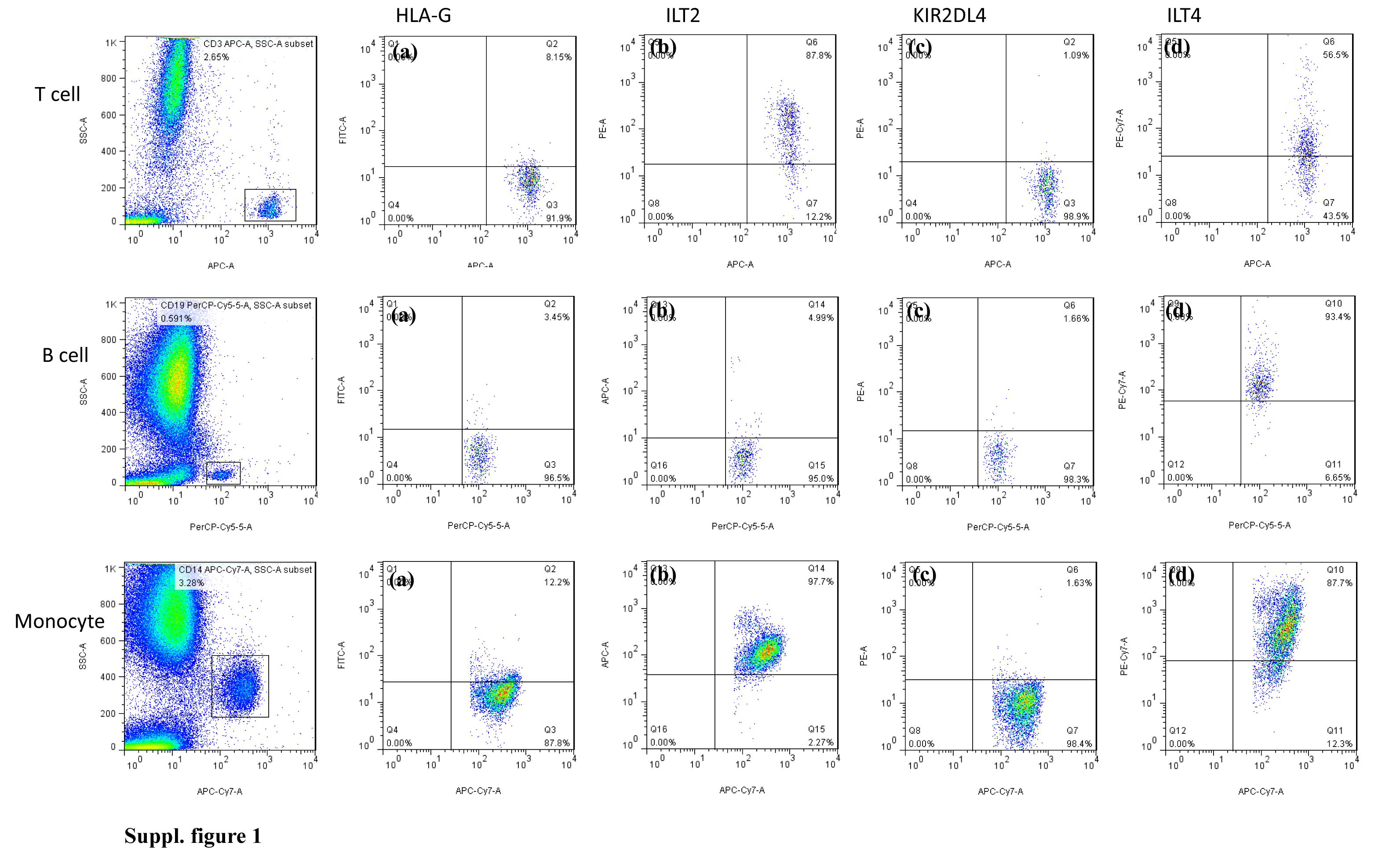

Supplement: Supplementary file 1 — Supplementary figure 1 [file CTI2-9-e1128-s001.tif]
